# Modeling salinity effect on rice growth and grain yield with ORYZA v3 and APSIM-Oryza

**DOI:** 10.1016/j.eja.2018.01.015

**Published:** 2018-10

**Authors:** A.M. Radanielson, D.S. Gaydon, T. Li, O. Angeles, C.H. Roth

**Affiliations:** a International Rice Research Institute (IRRI), Los Baños, Philippines; b Commonwealth Scientific and Industrial Research Organisation (CSIRO), Brisbane, Australia; c School of Agriculture and Food Sciences, University of Queensland, Brisbane, Australia

**Keywords:** Genotype, Soil, Photosynthesis, Transpiration, Water

## Abstract

Development and testing of reliable tools for simulating rice production in salt-affected areas are presented in this paper. New functions were implemented in existing crop models ORYZA v3 and the cropping systems modelling framework APSIM. Field experiments covering two years, two different sites, and three varieties were used to validate both improved models. We used the salt balance module in the systems model APSIM to simulate the observed daily soil salinity with acceptable accuracy (RMSEn <35%), whereas ORYZA v3 used measured soil salinity at a given interval of days as a model input. Both models presented similarly good accuracy in simulating aboveground biomass, leaf area index, and grain yield for IR64 over a gradient of salinity conditions. The model index of agreement ranged from 0.86 to 0.99. Variability of yield under stressed and non-stressed conditions was simulated with a RMSE, of 191 kg ha^−1^ and 222 kg ha^−1^_,_ respectively, for ORYZA v3 and APSIM-Oryza, corresponding to an RMSE_n_ of 14.8% and 17.3%. These values are within the bounds of experimental error, therefore indicating acceptable model performance. The model test simulating genotypic variability of rice crop responses resulted in similar levels of acceptable model performance with RMSE_n_ ranging from 11.3 to 39.9% for observed total above ground biomass for IR64 and panicle biomass for IR29, respectively. With the improved models, more reliable tools are now available for use in risk assessment and evaluation of suitable management options for rice production in salt-affected areas. The approach presented may also be applied in improving other non-rice crop models to integrate a response to soil salinity − particularly in process-based models which capture stage-related stress tolerance variability and resource use efficiency.

## Highlights

• ORYZA v3 and APSIM-Oryza models were improved to account for salinity effects on rice production.
• Variability of soil salinity was represented by a simple linear relationship between salt concentration and electrical conductivity.
• The derived salinity parameters captured response differences between tolerant (BRRI Dhan47) and non-tolerant variety (IR64).
• An increase in salinity parameters of 5 % above the value for IR64 would result in a 3 % increase in simulated yield.

## 1 Introduction

Rice-based cropping systems are increasingly threatened by the effects of climate change because large portions of rice-growing areas are located in vulnerable regions (Masutomi et al., 2009). Particularly in the coastal zones of South and Southeast Asia, the source of more than 65% of global rice, sea level rise is among the main direct consequences of climate change leading to increased flood and salt intrusion into inland fresh water. In irrigated systems, availability of fresh water becomes limited, leading to many areas being abandoned to fallow, thus significantly reducing rice production and food security for these regions (Wassmann et al., 2009).

Rice is considered as a salinity sensitive crop (Maas and Hoffman, 1977; Flowers and Yeo, 2004, Munns and Tester, 2008). The critical salinity level resulting in 50% yield loss was estimated to be around 6.9 dS m^−1^ for rice (Van Genuchten and Gupta, 1993). However, crop damage may vary with salt stress timing and the corresponding crop growth stage (Lutts et al., 1995; Khan et al., 1997; Zeng and Shannon, 2000). Salinity tolerance is a complex characteristic involving a number of component traits such as sodium uptake and compartmentalization. Minimization of salt uptake by the roots and partitioning salt at the tissue and cellular level reduced buildup of Na^+^to toxic concentrations for transpiring leaves and actively growing tissues (Munns, 2005; Munns and Tester, 2008). Genotypic variability in salinity tolerance in rice is mainly due to differing abilities in excluding Na^+^ from the shoot (Lee et al., 2003; Ren et al., 2005; Platten et al., 2013). Higher Na^+^ exclusion and increase of K^+^ absorption helps tolerant plants to maintain low Na^+^/K^+^ in the shoot, conserving photosynthetic functioning and tissue growth (Gregorio and Senadhira, 1993; Yamane et al., 2009). Furthermore, salinity tolerance is variable with growth stage, with seedling and ‘panicle initiation to flowering’ being the most sensitive periods (Moradi et al., 2003).The use of tolerant varieties can alleviate salinity problems in salt-affected rice-growing areas but this must be combined with other crop management technologies such as a suitable cropping schedule (Plaut et al., 2013).

It is expensive to experimentally quantifying the combined effects of tolerant varieties and adaptive management practices (such as irrigating with saline water) on chemical leaching and on yield for every possible situation. Similarly, obtaining viable management adaptation options purely through field experiments is costly and time-consuming. Also, field experiments alone provide limited information on long-term variability in system performance. They are influenced by the site-specific environmental and management conditions imposed on treatments usually performed over a limited number of seasons. Well-tested computer simulation models that integrate the effects of crop management on stress-affected conditions (as in a salt-stressed environment), can be used in extrapolating experimental results to include long-term temporal effects of climate and to account for various management practices and soil types (Matthews et al., 1997; Bannayan et al., 2005; Phogat et al., 2010;Laborte et al., 2012; Li et al., 2013). In conjunction with experiments, we can then develop and evaluate potential adaptive strategies. The utility of this approach requires however that the models can adequately reproduce and represent the system processes and performance associated with those strategies.

Modeling initiatives have previously integrated plant responses to salinity at the crop level such as those reported for wheat, bean, and tomato (Adiku et al., 2001; Karlberg et al., 2006; Corwin et al., 2007, Webber et al., 2010). A few studies have been undertaken on rice (Tuong et al., 2003, Phogat et al., 2010, Mondal et al., 2015). Most of these models were based on an empirical description of the effects of salinity using the Maas and Hoffman equation (1977) and have the advantages of simplicity and reliability in yield loss estimation. Detailed representations of soil salinity are also available in many useful and recognised soil models such as SWAP (van Dam et al., 2008), but crop responses are limited to the reduction of water uptake, mimicking the water deficit effect of salinity (Castrignano et al., 1998, Ferrer-Alegre and Stockle, 1999, Hochman et al., 2007). Plant responses were assumed to be controlled by average soil salinity within the root zone, which is not always true (Rhoades and Merrill, 1976). Furthermore, such empirical approaches are limited to the experimental conditions under which they were developed and have limitations in capturing the temporal variability of the stress (Singh et al., 2006; Crescimanno and Garofalo, 2006; Corwin et al., 2007).

The water productivity approach is widely adopted where water productivity decreases with salinity increase (e.g. Castrignano et al., 1998, Homaee et al., 2002, Smets et al., 1997, Jiang et al., 2011, Adiku et al., 2001, Phogat et al., 2010, Tuong et al., 2003). Some authors have reported that a reduction in crop yield due to reduced water uptake induced by soil salinity is valid for crops with a maximum water productivity of 4.21 kg m^−3^, up to a salinity level of 8 dS m^−1^ (Van Dam and Malik, 2003). Considering only limited water uptake might lead to overestimation of growth in relation to transpiration, particularly when the plants have invested assimilates into counteracting the adverse effects of salinity − for instance the ion pumps required for recirculation and compartmentalization of toxic ions (Asch and Wopereis, 2001; Ismail et al., 2007). In contrast, because the plant does respond to increased salinity stress in ways other than by reducing its transpiration through stomata closure, simulating salinity effect only by an increase in respiration (as in the CoupModel) may underestimate the decrease of growth (Karlberg et al., 2006).

Available rice models are then limited in accounting for salinity effect on growth and yield under dynamic salt-affected conditions. Representation of the salinity effects with a function accounting for interactions between genotype, environment and management has then to be considered. We assumed that an approach integrating algorithms to describe both the osmotic stress component of salinity (by affecting soil water uptake) and the varietal tolerance to salinity into an existing robust rice simulation model could effectively capture the complexity of rice crop responses to salinity.

Radanielson et al. (2017) proposed a new function describing rice crop responses to salinity, accounting for the variability of responses in biomass production processes (namely transpiration and photosynthesis) among different rice varieties. The function requires only two genotype-dependent parameters which simplifies parameterization and allows flexibility for model users investigating parameter variation within the genetic background of the considered variety. These parameters were related to the ability of the crop to produce biomass and to account mainly for the effect of salinity during the vegetative stage. The rice model ORYZA v3 (Bouman et al., 2001, Li et al., 2017) is considered the most exhaustive representation of available knowledge in rice (IRRI, 2017). It has been widely used to explore crop limitations and opportunities for increasing rice productivity under different environmental conditions and in various rice-growing areas (Aggarwal et al., 1997; Das et al., 2007; Feng et al., 2007; Artacho et al., 2011; Vaghefi et al., 2011; Sudhir-Yadav et al., 2011, Sudhir-Yadav et al., 2012; Laborte et al., 2012; Li et al., 2013). The model has been widely employed by various studies in terms of physiological analysis (Kropff and van Laar, 1994), temporal, spatial scale simulation (Krishnan et al., 2007, Van Wart et al., 2013) and management optimization (Belder et al., 2007; Xue et al., 2008).

A version of this model is also integrated into the broader cropping systems model, APSIM (Holzworth et al., 2014; Keating et al., 2003; Gaydon et al., 2012a, Gaydon et al., 2012b, Gaydon et al., 2017). The APSIM framework allows a detailed specification of farmer management practices, decision-trees, and the simulation of associated soil water and salinity dynamics, together with the interactions between rice and other crops, allowing for a much broader assessment of cropping system performance than can be provided by ORYZA V3. Furthermore APSIM offers an opportunity to simulate the soil salinity dynamics based on the interaction between soil water availability, movement, and soil water solute concentration. These variables presented changes with the genotype (nutrient uptake and lower limit of soil water extraction), environmental (soil type, climatic conditions) and management factors (imposed irrigation management and irrigation water salinity). The model ORYZA uses a linear interpolation function to represent daily values of soil salinity from inputs of soil salinity measured at given intervals.

We present here then the improvement of the rice model ORYZA v3 and the cropping system model APSIM to account for salinity response in rice, together with validation in representing affected production systems. Algorithms describing rice crop responses through physiological processes related to water deficit and ion toxicity were incorporated i.e. the function determined by the two parameters, namely, 1) the salinity threshold and 2) the rate of growth reduction as salinity increases beyond the threshold.

The improved models were parameterised and calibrated with experimental data and their performance in representing rice production under salinity was subsequently validated with independent field experimental data.

## 2 Materials and methods

### 2.1 General descriptions of models used

**Fig. 1:**
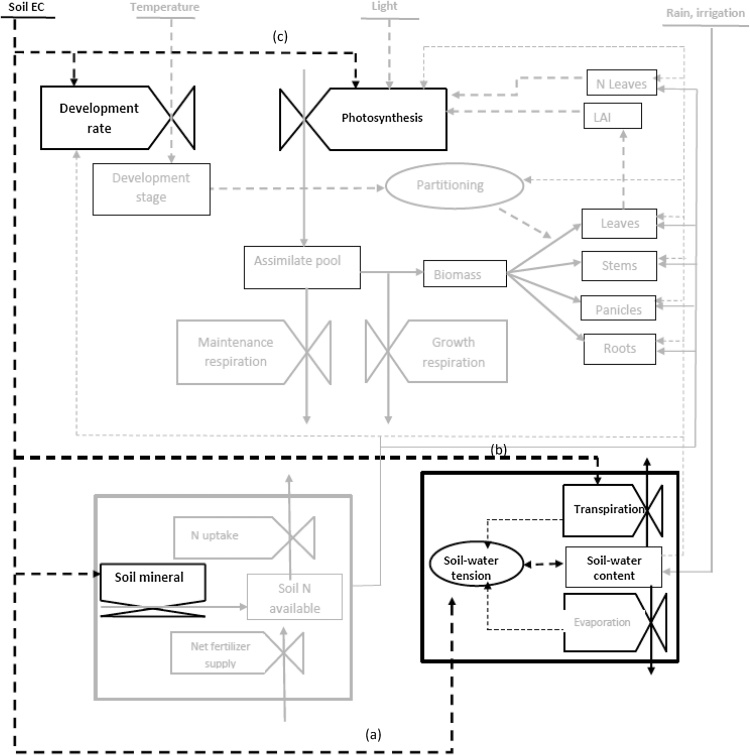
Diagram representing the ORYZA v3 model and the flow of mass and information between its different modules. The tied boxes represent process rates; the boxes represent state variables, and the circles, the intermediate variables. The dashed lines represent flow of information and the continuous lines represent flow of mass. The black lines and text indicated the processes which representation was modified with the new functions accounting for the salinity effect. LAI, leaf area index; N, nitrogen. (a)–(c) indicated the processes affected by the salinity effect namely soil solute content changing soil osmotic potential component and soil water tension (a), transpiration rate (b) and leaf photosynthesis (c).

The ORYZA v3 rice crop model (Li et al., 2017) fully inherited all the features of the previous versions of the ORYZA2000 rice model (Bouman et al., 2001). It is an ecophysiological model simulating daily growth and development of rice, as well as soil water balance (Bouman et al., 2001; Bouman and van Laar, 2006). It is dynamically driven by photosynthesis on a daily time-step basis, with a tipping-bucket water balance approach (Fig. 1, Bouman et al., 1996; Bouman et al., 2001, van Ittersum et al., 2003). It has evolved from an explanatory model with descriptive elements such as phenology and biomass partitioning (Bouman et al., 2001; Bouman and van Laar, 2006; Sheehy et al., 2006; Tang et al., 2009; van Oort et al., 2011) to a comprehensive model with mechanistic processes such as root growth, water and nitrogen uptake, and soil carbon/nitrogen dynamics (IRRI, 2017, Li et al., 2017, Fig. 1). Drought is represented by effects on leaf expansion, leaf rolling, leaf senescence, photosynthesis, assimilate partitioning, root growth, and spikelet sterility. All simulations associated with drought effects are accomplished by multiplying drought-stress factors (0–1) with potential production (Boling et al., 2007). Input requirements of the model are geo-coordinates of the site studied, daily weather data (maximum and minimum temperature, rainfall, solar radiation), plant density, date of transplanting or seed sowing, variety-specific parameters defining morphological and physiological characteristics such as crop development rate constants, partitioning functions, and a stem carbohydrate translocation factor. A large number of parameters are set in the model, of which most are default values for rice. Parameters reflecting individual varietal genetic characteristics such as development rates, partitioning factors, relative leaf growth rate, specific leaf area, leaf death rate, and fraction of stem reserves require high-quality experimental data to calibrate.

The APSIM model is a cropping simulation framework capable of simulating the performance of diverse cropping systems, with rotations, fallow periods, crop and environmental dynamics, while considering detailed farmer management and farmer decision-trees specified by the user (Holzworth et al., 2014; Keating et al., 2003; Cichota et al., 2013). It has been recently enhanced to simulate complex farming systems that involve rice-based rotations. The APSIM rice model is known as APSIM-Oryza. The APSIM-Oryza model integrated all components of rice growth and yield formation from the ORYZA2000 model (V2.13) and has updated algorithms and processes describing anaerobic soil processes and pond biogeochemistry (Gaydon et al., 2012a, Gaydon et al., 2012b). APSIM-Oryza has been demonstrated to adequately represent the production of rice varieties in rotation with other non-flooded crops and pastures over long-term periods, along with water and nutrient dynamics in the field (Gaydon et al., 2012a, Gaydon et al., 2012b, Gaydon et al., 2017).

### 2.2 Simulating crop responses to salinity in ORYZA v3 and APSIM-Oryza

Two components of crop salinity response (osmotic stress and ion toxicity stress) were added as new elements in the model modification. Osmotic stress is taken into account through an equation converting daily soil salinity into daily soil osmotic potential, resulting in a daily decrease in water available for uptake by the crop (Eq. (1), Fig. 1-a). The osmotic potential of the soil solution is increasing in proportion to the soil solution salt concentration, as per the equation below (FAO, 2013):

(1) OSKPA=40.55EC

where *OSKPA* is the soil osmotic potential and *E*C is the soil electrical conductivity (dS m^−1^).

Soil osmotic potential is then added to the matric potential determined by soil water balance module on a daily basis. Osmotic potential is assumed to be limited only by soil EC, which is driven by soil salt content expressed as chloride ion (Cl^−^) content.

**Fig. 2:**
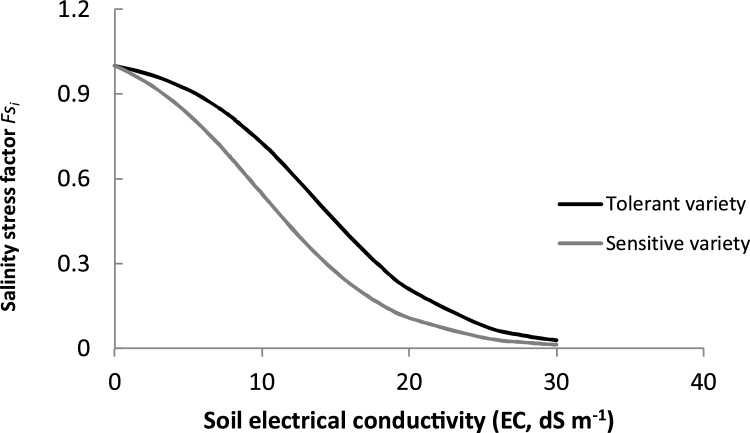
Example of salinity stress factor curves for varieties with different salinity tolerances (Adapted from Radanielson et al., 2017). *FSi* is the salinity stress factor, where *i* is the considered process (photosynthesis rate or transpiration rate). Salinity stress factor curves are mainly determined by two parameters, the slope of decrease in the process rate at the inflection point (parameter *a*) and the critical value of salinity for 50% of loss of the process rate to occur (parameter *b*). In this example, the tolerant variety has parameters (*a)* and (*b*) equivalent to 0.22 and 7.83 respectively. The sensitive variety has parameters (*a*) and (*b*) equivalent to 0.22 and 13.69 respectively.

Ion toxicity stress is assumed to be driven by the accumulation of Na^+^ into the shoot up to a critical value, a process which depends on the environment (level of soil solution salinity, the duration of the stress, climatic conditions such temperature and relative humidity) and the genotype (tolerance level of the variety and its phenology). In the model, we capture the effect of the stress duration through the integration, of the daily stress effect during the plant exposure over the period of crop growth. The dynamics of plant water availability varies with climatic conditions and soil water osmotic potential and matric potential. Rice crop responses to salinity are characterized according to Radanielson et al. (2017) which reports on the variability of leaf transpiration rate (Fig. 1-b) and leaf photosynthesis (Fig. 1-c) responses to salinity among different rice varieties. Salinity effect on the latter processes was represented using a logistic function − an approach which is commonly used to represent the abiotic stress factor on plant growth (Karlberg et al., 2006; Casadebaig et al., 2008). Daily maximum plant photosynthesis rate *(pn)* and transpiration rate *(tr)* were then multiplied by the stress factor. The stress factor has two parameters that are considered genotype-dependent (Eq. (2), Fig. 2):

(2) $$FS_{i}={\left(1+e^{a_{i}\left(EC-b_{i}\right)}\right)}^{-1}$$

where *FS_i_* is salt stress factor; *i*, the considered process i.e. photosynthesis rate *(pn)* or transpiration rate *(tr)*; EC, soil electrical conductivity (dS m^−1^); *a,* slope of the decrease of the process rate at the inflection point at 50% of maximum rate; and *b*, critical value of salinity for 50% of loss of the process rate.

Daily actual transpiration rate under salinity (Fig. 1-b) was computed as the potential transpiration rate under non stressed conditions multiplied by the actual drought stress factor (FTSW, fraction of transpirable soil water) driven by the variation of soil water potential (Wopereis et al., 1994, Li et al., 2017) and by the salinity factor, the variation of which is driven by soil salinity expressed in soil electrical conductivity terms (Eq. (2)). Actual soil water potential (Fig. 1-a) was computed as the sum of osmotic potential computed in Eq. (1) and the soil matric potential. The effect of salinity on transpiration rate is then determined from the two components: osmotic stress and the ion toxicity. The salinity factor is used as an indicator for accumulated Na^+^ in the shoot representing the effect of potential ion toxicity on transpiration regulation.

Leaf photosynthesis under salinity (Fig. 1-c) was computed as the maximum leaf photosynthesis under the potential conditions multiplied by the salinity factor, the variation of which is driven by soil salinity expressed in soil electrical conductivity terms (Eq. (2)). The reduction of leaf photosynthesis with the limitation of water uptake is considered in the model and associated to the reduction of transpiration by the drought factor defined in the original model (PCEW) (Wopereis et al., 1994, Li et al., 2017).

Within the salinity factor *FS_i_,* the parameter *(b),* the level of salinity with 50% of loss of transpiration and photosynthesis rate, represents the salinity tolerance of the plant. It represents variability among rice crop genotypes and is likely correlated to the amount of Na^+^ accumulated in the shoot (Radanielson et al., 2017). The parameter *(b)* value increases with increased varietal salinity tolerance. The parameter (*a)*, which is the slope of the curve at its inflection point, represents the rate of decrease during a linear phase of the process beyond a critical level of salinity, triggering significant decrease in the plant growth. The parameter (*a)* is related to the salinity resilience of the genotype. It represents the ability of the rice crop to maintain and adjust its growth, limiting the irreversible effect of the Na^+^ toxicity. The value of (*a)* decreases with an increased resilience of the variety. The same crop parameters related to salinity tolerance were used for both models since their salinity effect representation utilize the same equation as indicated in Eq. (2) and as estimated by Radanielson et al., 2017 (Table 2).

#### 2.2.1 Salinity crop parameters estimation

For the three varieties used (IR29, IR64 and BRRI Dhan47) responses to salinity were extensively characterized in a greenhouse study (Radanielson et al., 2017). The varieties were grown in a greenhouse under a gradient of salinity levels from non-stressed control conditions, to light (4 ds m^−1^), mild (8 dS m^−1^), and severely-stressed conditions (higher than 12 dS m^−1^). Leaf transpiration rate and leaf photosynthesis was measured in addition to the accumulated crop biomass produced. Leaf transpiration and leaf photosynthesis responses to salinity were fitted using Eq. (2) to estimate the parameters *(a)* and (*b)*. The value of parameter (*b*) varied significantly between the processes, in contrast to parameter (*a)* which did not vary. We used values as reported by Radanielson et al., 2017 (Table 2). These two parameters were then defined as genotype-dependent and assumed constant under the environmental conditions of the field experiments conducted for evaluating the performance of the models.

#### 2.2.2 Simulating dynamics of soil salinity with APSIM

In APSIM, soil salinity dynamics are simulated using the APSIM-Solute module in conjunction with APSIM-Soilwat. A computation of soil layer-based dynamics of different ions is performed as a function of their specified solubility and transportability with soil water. APSIM simulates the dynamics of specified solutes within the individual layers of the soil profile (in kg ha^−1^ or ppm) (Probert et al., 1998; Huth et al., 2010). The crop is able to respond to these, depending on which soil layers its roots are exposed to (Lynch and Brown, 2001; Homaee et al., 2002). Soil salinity is assumed to be reflected by the distribution and amount of chloride ions in the soil. The amount of chloride applied via irrigation water (ranging from fresh to saline) was estimated by its mass molar equivalent in terms of NaCl. The equivalent amount of chloride was estimated and used as input with the modality of irrigation given in the experimental file for model simulations. APSIM then simulates the resultant saline profile within the soil on a daily basis using the concentration of salt of the irrigation water defined in the cropping manager script in the model. In contrast, direct measured daily soil salinity data is required as inputs when using the ORYZA v3 rice model. When daily data are not available, measurements at regular intervals are linearly interpolated by the model, to compute daily values.

### 2.3 Model evaluation

Data collected from field experiments performed at the International Rice Research Institute (IRRI) in Los Baños, Philippines and an on-farm experimental station in Infanta Quezon, Philippines were used for model calibration and subsequent validation (Table 1). Salinity parameters for the genotypes used, IR64, IR29 and BRRI Dhan47 were not calibrated, but were estimated from greenhouse experiments detailed in Radanielson et al. (2017). These are summarized in Table 2.

**Table 1:** Climatic conditions during the rice growing seasons of 2012–2014 for the Experiments 1–4.

Experiment	Season	Start and end of the season	Temperature (^°^C)		Total Rainfall mm	Total radiation MJ m^−2^
			Minimum	Maximum		
Expt.1	Dry season	30 November 2011–19 March 2012	24 ± 0.7	29 ± 1.4	434.1	1541.87

Expt.2	Wet season	June 6–19 September 2012	25 ± 0.9	31 ± 1.7	1115.1	1543.06

Expt.3	Dry season	26 January–13 May 2013	23 ± 1.1	31 ± 2.3	768.2	1830.24

Expt.4	Dry season	15 January–13 May 2014	23 ± 1.7	28 ± 2.0	389	2041.9

**Table 2:** Salinity response parameters of the genotypes, *a* is the slope of decrease in the process rate at the inflection point and *b* is the critical value of salinity for 50% of loss of the process rate. Values were derived from Radanielson et al. (2017) from greenhouse experiments and calibrated to the field crop responses.

Genotypes	Photosynthesis		Transpiration	
	*a_pn_*	*b_pn_*	*a_tr_*	*b_tr_*
IR64	0.21	11.17	0.21	7.83
IR29	0.31	11.08	0.31	6.83
BRRIdhan 47	0.21	15.15	0.21	12.30

#### 2.3.1 Field experiments

The field experiments conducted at the IRRI experiment station (121.15 E, 14.11 N) from 2011 to 2012 covered the dry (Expt.1, December 2011–March 2012) and wet (Expt.2, June–September 2012) seasons (Table 1). The experiments had a completely randomized block design with three replications using the genotype IR64. Each plot corresponded to a salinity treatment. The field experiments performed in the farm field at Infanta, Quezon, Philippines (121.41 E, 14.45N) covered two dry seasons (Jan–May) from 2013 (Expt.3) to 2014 (Expt.4) each had a split plot design. Each main plot corresponds to a salinity treatment while the subplot corresponds to variety, Three genotypes with contrasting salinity tolerance (IR29, IR64 and BRRI Dhan47) were used. They are characterized as sensitive, intermediate and tolerant to salinity, respectively. Seedlings were transplanted at 21 days after sowing (DAS) and received full irrigation with recommended N fertilizer application of 135–195 kg N ha^−1^, applied in four splits.

In all experiments, fields were maintained under continuous flooding and comprised a control plot irrigated with fresh water (0.8-1.4 dS m^−1^) and three saline treatments corresponding to salinity levels of 4 dS m^−1^ (3.2-4.8 dS m^−1^), 6 dS m^−1^ (5.4-6.1 dS m^−1^), and 12 dS m^−1^ (7.7-11.4 dS m^−1^). Salinity levels in each treatment plot were achieved and maintained by broadcasting salt in the ponded water at each irrigation event from 5 days after transplanting until 10 days before harvest.

Salinity levels at 15-cm depth were monitored daily in each plot with a 5TE sensor (Decagon Devices, USA) that records soil salinity, every 60 min. The measured values were stored on an EM50 data logger (Decagon Devices, USA). To manage salt application and maintain the target salinity levels in Expts 1 and 2, daily monitoring of soil solution EC was also performed using a hand-held EC meter that measured soil water in perforated PVC field tubes installed to a 15-cm depth. In expts 3 and 4, soil solution EC was also recorded; however, salt application was managed uniformly within each salinity treatment using a constant amount of salt applied at each irrigation event, disregarding the soil solution EC before irrigation.

An automated weather station (Davis Instruments Corp., California, USA) was installed near the field, allowing continuous recording of climatic data, including air temperature and relative humidity, rainfall and solar radiation. The weather information was formatted as input for ORYZA v3 and APSIM-Oryza using a daily value for minimum and maximum temperature, rainfall, vapour pressure deficit, and total daily radiation.

#### 2.3.2 Measurements

Crop phenology dates were recorded for panicle initiation, flowering and physiological maturity. Total above ground biomass and leaf area were also monitored by sequential sampling of plants at 28 DAS, 42 DAS, 50% flowering, grain filling and physiological maturity. Twelve hills were sampled from each replicate every sampling time. Final grain yield from each replicate was estimated at harvest over a sampling area of 5 m^2^.

#### 2.3.3 Crop model parameterisation and calibration

The phenological development rate parameters for the three varieties used were computed using the observed phenology dates from plots of each salinity treatment. Crop parameters for leaf growth and biomass allocation were derived from the control plot (irrigated with fresh water) in dry and wet seasons using the auto-calibration application of ORYZA v3, assuming non-limited environmental growth conditions. The statistical criteria for calibration were set to minimize the deviation of the simulation outputs against the observed values for variables: total aboveground biomass, grain yield, and leaf area index (LAI). The calibrated crop parameters used for ORYZA v3 were the same as those used for APSIM-Oryza.

In all simulations, APSIM also simulated the dynamics of soil salinity based on the management information specified, whereas ORYZA v3 used direct measurements of soil salinity recorded daily during crop growth as inputs to the model.

#### 2.3.4 APSIM soil salinity simulation initialization and inputs

Soil salinity was simulated using the Solute module of APSIM considering an initial non-saline soil salinity status. Soil salinity then varied as saline water was applied with the irrigation water, corresponding to an application of additional salt to the soil solution. The APSIM-Solute module used this amount of salt (corresponding to the salinity level of the irrigation water equivalent weight molar of chloride) in determining the soil solute balance on a layer-by-layer basis. Movement and diffusion of the chloride through the soil layers with soil water determined the concentration of chloride within the root profile, which was converted to soil solution salinity level using the equation below (3):

(3) SEC = [Cl]/640

where SEC is the soil electrical conductivity in dS m^−1^, [Cl], is the average soil layer concentration of chloride over the root profile depth.

The salinity of soil solution calculated is then used in the computation of soil osmotic potential following Eq. (1), and the factor of salinity stress applied to the models’ crop growth function for crop assimilation and transpiration following Eq. (2).

#### 2.3.5 Model validation

The ability of the models to represent the crop response to the salinity experienced was evaluated with data collected from the salt-treated plots.

Linear regression was used to compare paired data-points for measured and simulated above-ground biomass, grain yield, and LAI. The slope (α), intercept (β), and coefficient of determination (R^2^) of the linear regression between the simulated and measured values were determined. The model performance was evaluated using the Student’s *t*-test of means, assuming unequal variance P(t), the root mean squared error, RMSE, and the normalized root of the mean squared error (RMSE_n_, %) (Eq. (4)):

(4) $${{}_{RMSE}}_{n}=\frac{\sqrt{\sum_{i=1,n}{\left(S_{i}-O_{i}\right)}^{2}}}{n\;\;\mu }\times 100$$

where S_i_ and O_i_ are simulated and observed values, respectively, n is the number of pairs, and μ is the overall mean of the observed values.

A model reproduces experimental data best when 1) α, β, R^2^ are close to 1, 0 and 1 respectively, 2) the P(t) value is larger than 0.05 (indicating observed and simulated data are the same at the 95% confidence level), and 3) the RMSE between simulated and observed values is similar or less than the standard deviation of the experimental measurements across replications.

The index of model agreement (ID) was also used as a measure of model performance, calculated as indicated in Eq. (5) as proposed by Willmott (1981).

(5) $$ID=1-\left[\frac{\sum_{\text{i}=1}^{n}{\left({\text{S}}_{\text{i}}-{\text{O}}_{\text{i}}\right)}^{2}}{\sum_{\text{i}=1}^{n}{\left(|{{\text{S}}^{\prime }}_{\text{i}}\left|-|{{\text{O}}^{\prime }}_{\text{i}}\right|\right)}^{2}}\right]$$

where S_i_ and O_i_ are simulated and observed values, respectively; S’_i_ and O’_i_ are the difference between the simulated and observed values and the overall mean of the observed values; *n* is the number of pairs.

A value of ID being close to1 is desirable and indicates a good agreement between the simulated and observed values.

#### 2.3.6 Model sensitivity analyses

Sensitivity analyses were performed to understand the effect of varying the parameters in Eq. (2) on the model simulation outputs. The *a_pn_* and *a_tr_* (slopes of decrease in photosynthesis and transpiration rates), and *b_pn_* and *b_tr_* (critical values of salinity at 50% loss in photosynthesis and transpiration) were investigated in the sensitivity analysis.

These four parameters were combined with a change of ±one to six times their standard deviation observed in the greenhouse studies (Radanielson et al., 2017, Table 2). Each parameter was changed individually, while the other parameters were held constant. The virtual combinations were used in simulations covering the four salinity levels in the field experiments used for calibration and validation, using historical weather data of Los Baños, Philippines, from 2000 to 2012. In the ORYZA model, we assumed that the dynamics of annual soil salinity during the 12 years was constant. Analysis of variance (ANOVA) was done on the simulated yield, total biomass, and water productivity using the R software. Water productivity was calculated as the ratio of the simulated total transpired water to simulated yield (kg ha^−1^ mm^−1^). Transpired water was computed by the water balance of the model as described in Bouman et al. (2001). The contribution of each parameter’s variation to the variability of the model outputs was quantified using variance component analysis performed with a linear mixed-effects model using the package lme4 (Bates et al., 2008) in R software and expressed in relation to the coefficient of variation.

Genotype (represented by a combination of *a_pn_*, *a_tr_* and *b*) was used as random effect and the environment (represented by the climatic conditions variability) as fixed effect.

## 3 Results

### 3.1 Weather and soil salinity conditions for the experiments

Climatic conditions during the experiments 1–4 reflected average maximum (28–31 °C) and minimum temperatures (23–24 ^°^C), which provided non-stressed conditions for the rice crop (Table 1). Season rainfall was also sufficient with distinct wet and dry season with total rainfall ranging from 389 to 1115.1 mm (Table 1).

**Fig. 3:**
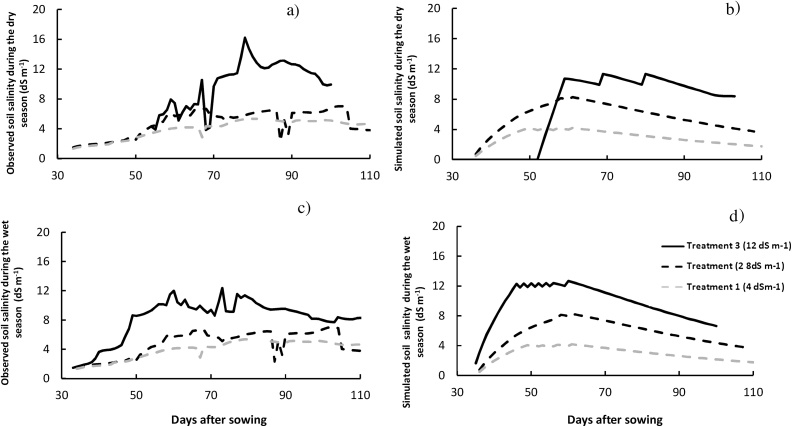
Measured soil salinity using 5TE sensors (Decagon, USA) and simulated using APSIM-Oryza during the dry season 2012 in experiment 1 (a, b) and during the wet season 2012 in experiment 2 (c, d). Dashed gray lines at 4 dS m^−1^, dashed black lines at 8 dS m^−1^, and black solid lines at 12 dS m^−1^.

The soil salinity levels achieved in the experiments showed daily variability for each target salt level. The gradients among treatment levels were generally maintained. APSIM was able to simulate soil salinity dynamics satisfactorily (Fig. 3). The calculated error was within the range of the experimental standard deviation, with an RMSE_n_ of about 34.0%, (approximately half the coefficient of variation of the measured values). The ID was about 0.86, covering more than 50% of the observed values’ variability as indicated by R^2^ values ranging from 0.55 and 0.90 (Fig. 3a, Table 3). Soil salinity simulation for the experiments 1 and 2 (2012–2013) presented larger errors than the simulation for the experiments 3 and 4 (2013–2014) (Fig. 3a and b). The larger errors in experiments 1 and 2 with the APSIM simulations were related to the rules specified in simulating the imposed salinity treatments to reach higher stress at 12 dS m^−1^. In the observed values from the experiments, soil salinity reached up to 14 dS m^−1^ whereas in the model the application of saline water was limited when the soil salinity at 15 cm depth reached 12 dS m^−1^ of irrigation. The relationship between amount of salt applied and irrigation water salinity therefore presented reduced accuracy beyond the salinity level of 8 dS m^−1^. In contrast in experiments 3 and 4, a regular frequency of irrigation was observed using a constant amount of salt applied which imposed different levels of stress between the sets of experiments as indicated in Section 2.2.

**Table 3:** Statistics on the observed and simulated data for model performance evaluation.

Model	Variables	*n*	X_obs_ (sd)	X_sim_ (sd)	P(t^*^)	β	α	R^2^	RMSE	RMSE_n_ %	ID
ORYZA v3	Above-ground biomass (kg ha^−1^)	*25*	3795 (2437)	4217 (2949)	0.58	−243	1.17	0.94	694	18.28	0.97
	Yield (kg ha^−1^)	*25*	1282(1502)	1358 (1607)	0.86	−3	1.06	0.98	191	14.88	0.99
	LAI	*23*	1.39 (1.17)	1.64 (0.89)	0.39	0.75	0.64	0.69	0.47	34.21	0.88
APSIM-Oryza	Above-ground biomass (kg ha^−1^)	*25*	3795 (2437)	3864 (3142)	0.93	−934	1.26	0.95	608	16.02	0.97
	Yield (kg ha^−1^)	*25*	1282 (1502)	1502(1723)	0.75	−34	1.13	0.98	222	17.30	0.98
	LAI	*23*	1.39 (1.17)	1.51 (0.90)	0.67	0.57	0.68	0.76	0.42	30.29	0.91
	Soil EC (dS m^−1^)	*346*	6.09 (3.02)	6.48 (3.13)	0.98	1.77	0.77	0.56	2.07	34.01	0.86

X_obs_, mean of measured values; X_sim_, mean of simulated values; sd, standard deviation; *n*, number of data pairs; P(t^*^), significance of Student’s paired *t*-test assuming non-equal variances; α, slope of linear regression between simulated and measured values; β, y-intercept of linear regression between simulated and measured values; R^2^, square of linear correlation coefficient between simulated and measured values; RMSE, absolute root mean squared error; RMSE_n_, RMSE normalized by X_obs_ in percentage; ID, Index of modelling agreement; LAI, leaf area index dynamic value during the crop growth; Soil EC, soil electrical conductivity (dS m^−1^).

### 3.2 Model performance

ORYZA v3 and APSIM-Oryza were initially calibrated under non-stressed conditions and then validated under salt-stressed conditions. The evaluation of both models was then performed in terms of their overall ability to simulate the observed values under different salt-stressed conditions.

The models’ accuracy in simulating rice crop growth through LAI, aboveground biomass, and rice yield was evaluated using RMSE and RMSE_n_ (Table 3). The RMSE between observed and simulated values was within the range of the standard deviation of the observed values (Table 3), indicating that the models were simulating the variables of interest within the bounds of experimental uncertainty, which is closest to reality that any model can achieve (Gaydon et al., 2017).

**Fig. 4:**
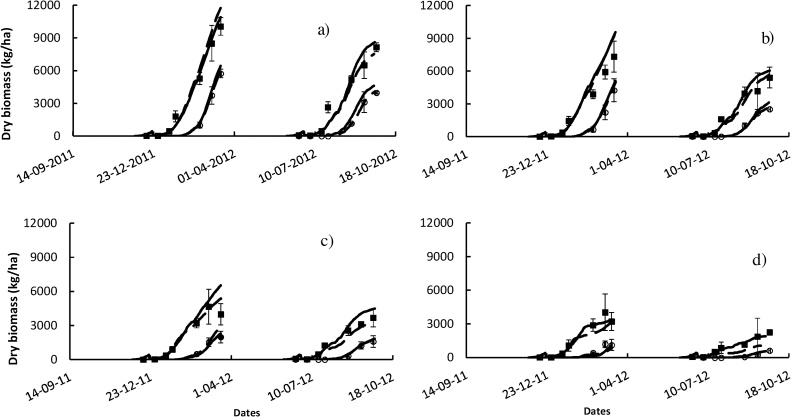
Simulated and observed values for biomass and grain yield under four different soil conditions: a) non-saline, b) 4 dS m^−1^, c) 8 dS m^−1^, and d) 12 dS m^−1^. Black lines represent outputs from APSIM-Oryza simulations; dashed black lines represent outputs from ORYZA v3; square points represent the observed above-ground biomass values from the 2012 wet and dry season experiments (Expt.1& 2) at IRRI, Los Baños, Philippines, and the open circle points represent the observed grain yield. Error bars are standard deviation of the mean.

Compared with APSIM-Oryza, the model ORYZA v3 demonstrated higher accuracy for grain yield with RMSE_n_ of 14.9%. The values of RMSE between simulated and observed grain yield were 191 kg ha^−1^ and 222 kg ha^−1^_,_ for ORYZA v3 and APSIM-Oryza, respectively. Simulations were able to represent 98–99% of the observed values, suggesting a good calibration of both models in yield simulation under non-stressed conditions (Table 3, Fig. 4).

Both models were able to simulate aboveground biomass acceptably, with RMSE_n_ about 16.0% and 18.3%, respectively, for APSIM-Oryza and ORYZA v3 (Table 3). Both models were able to represent the variability of observed above-ground biomass with an ID of 0.97.

Both models presented similar trends in capturing the effect of the different gradients of salinity on rice biomass production and yield. Under the lowest level of salinity (4 dS m^−1^), simulations were able to represent the light salinity stress compared with non-stressed conditions (Fig. 4a and b).

Under the mild stress level about 8 dS m^−1^ and the higher level of 12 dS m^−1^, the simulation outputs did not present any significant difference between the two models illustrating a consistent agreement with the observed values (Fig. 4c and d).

ORYZA v3 overestimated the salt effect on aboveground biomass production compared with simulations of APSIM-Oryza for the stressed condition at 12 dS m^−1^ (Fig. 4). These observations were attributed to the difference in values of soil electrical conductivity considered by the two models. In fact, the simulated soil salinity at 12 dS m^−1^ by APSIM-Oryza presented no values higher than 12 dS m^−1^, whereas the soil salinity measured and used as input for ORYZA v3 reflected values up to 14 dS m^−1^, implying higher effects of salinity in the simulations using ORYZA v3 (Fig. 4).

**Fig. 5:**
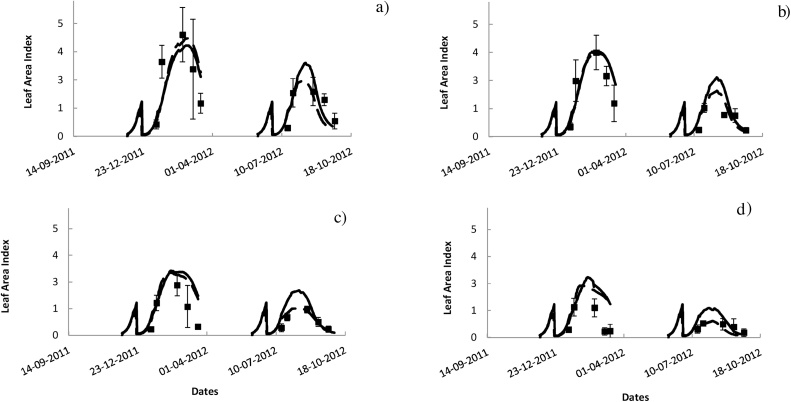
Simulated and observed leaf area index under four different soil conditions. a) non-saline, b) 4 dS m^−1^, c) 8 dS m^−1^, and d) 12 dS m^−1^. Black lines represent outputs from APSIM-Oryza simulation, black dashed lines represent outputs from ORYZA v3, square points represent the observed leaf area index (LAI) values from the 2012 dry and wet season experiments (Expt.1& 2) at IRRI, Los Baños, Philippines. Error bars are standard deviation of the mean.

The ability of ORYZA v3 to simulate most of the variability in LAI was similar to that of APSIM-Oryza with RMSE_n_ of 34.2% and 30.3%, respectively (Table.3). The weakness of the models in simulating dead leaf biomass was the main reason for the underestimated LAI. Nonetheless, the differences between simulated and observed LAI were within the range of the experimental uncertainty, represented by the standard deviation of the observed values (Fig. 5, Table 3).

In summary the modified models ORYZA v3 and APSIM-ORYZA were significantly improved in their ability to simulate rice crop growth and yield under salinity. Simulations using the original models overestimated rice growth and yield with an error of more than 100% (Supplementary Fig. 1 & Table 1). In contrast simulations using the modified models presented reduced error with RMSE_n_ within the coefficient of variation of the observed data (e.g 26.6–31.7%, Supplementary Table 1). The RMSE value was reduced using the modified models by more than 70% compared to the RMSE of the simulations using the original models (for instance between original and modified APSIM-ORYZA simulations, RMSE was reduced from 2704 kg ha^−1^ to 723 kg ha^−1^). Apart from the error, the improved models additionally explained an extra 23–33% of the variation between treatments (for example, R^2^ increased from 0.65 to 0.98 for prediction of WSO; 0.72–0.95 for WAGT; Supplementary Table 1)

### 3.3 Model evaluation in representing genotypic variability in rice crop responses to salinity

**Fig. 6:**
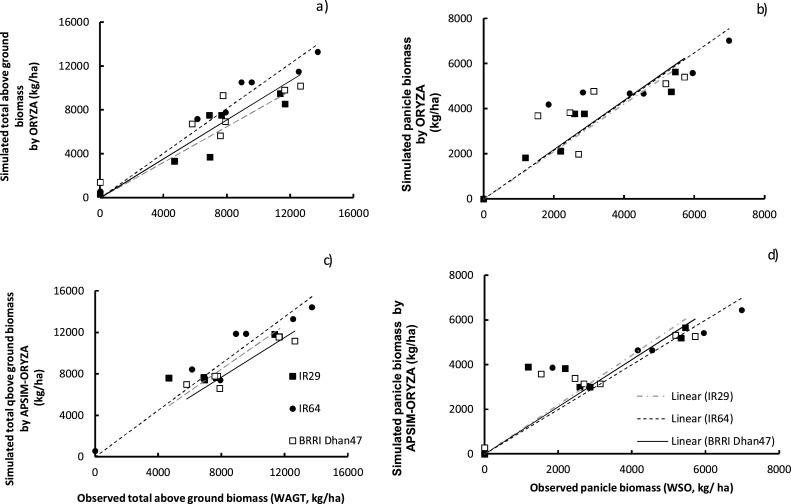
Simulated above-ground biomass and panicle biomass by the ORYZAv3 model (a, b) and by the APSIM-Oryza model (c, d) versus observed data for three contrasting varieties growing in salt stressed conditions. The observed data presented are average measurements from 3 replications in experiments 3 & 4 for each variety. Simulated data are obtained from the ORYZA v3 model (a, b) and APSIM-Oryza (c, d), considering salinity conditions of the experiments 3 & 4. Lines present the linear relationship between simulated and observed values with intercept at 0.

The models’ ability to represent genotypic variability in rice crop response to salinity was evaluated using the data observed from experiments 3 and 4 performed at Infanta. Simulated total above-ground biomass and yield presented an acceptable level of agreement with the data observed from these experiments (Expt.3 & 4) for the three studied genotypes (Fig. 6). The models achieved acceptable accuracy in capturing variability among seasons, sites and among varieties in representing salinity effect on rice total above ground biomass (RMSE_n_ ranging from 13.3 to 25.7%) and grain yield (22.1 to 39.9%, (Table 4)). Simulation under high salinity stress (lower observed values) presented underestimation with higher estimates with the model APSIM-Oryza. Variation between the two models outputs was partly explained by the underestimation of the soil salinity simulated by APSIM (RMSE_n_ of 30–34%, Table 4).

**Table 4:** Statistical parameters of the comparison between simulated and observed data for the three varieties IR29, IR64 and BRRI Dhan 47 using the modified ORYZA v3 and APSIM-ORYZA model. R^2^, coefficient of determination; RMSE_n_, Root mean square error normalized by the mean of the observed measurements (as percentage).

Model	Variety	Total above ground biomass (WAGT, kg/ha)			Dry weight of organ storage (WSO, kg/ha)		
		Slope	R^2^	RMSEn (%)	Slope	R^2^	RMSEn (%)
ORYZA V3	IR29	0.81	0.84	25.7	1.05	0.87	22.1
	IR64	1.01	0.94	11.3	1.08	0.68	29.2
	BRRI Dhan47	0.89	0.77	21.8	1.09	0.57	36.9
APSIM-ORYZA	IR29	1.11	0.86	21.1	1.11	0.55	43.1
	IR64	1.12	0.92	20.8	1.00	0.81	22.5
	BRRI Dhan47	0.96	0.90	13.3	1.05	0.69	28.1

Total above ground biomass presented better fit than the yield. Model simulation could be improved with a more detailed representation of the salinity effect on yield formation, for instance the use of an additional salinity stress factors on biomass partitioning, rice crop phenology and the spikelet sterility. These effects of salinity would be more related to the tolerance of the rice plant to salinity during the reproductive stage.

### 3.4 Model sensitivity analysis

The variability of the parameter related to rice transpiration responses to salinity *b_tr_* has a significant effect on the simulated rice yield variability (p < .001). This was also observed in the different salt-stressed environments considered. A change in the parameter *b_tr_* of about 5% will result in an increase of yield by about 3% compared with the reference genotype with a *b_tr_* of 7.83. However, the parameter related to photosynthesis *b_pn_* did not present a significant effect (p < .05), suggesting that the model outputs were not sensitive to variation of this parameter within the range of salinity levels tested. Variability of climatic conditions within the year explained more than half of the variation in yield and aboveground biomass but only contributed to 5% of the variability in water productivity.

The variation of the critical value for rice plant transpiration response to salinity *b*_tr_ illustrated a higher contribution to the variability in above-ground biomass and yield compared with the parameters related to the decreased rates of photosynthesis and transpiration (a_pn_, a_tr_). Variation in the latter parameters represented higher contribution to variability in water productivity, 65%.

In the range of variation of the critical value for photosynthesis responses *b*_pn_, no significant effect was observed on yield variability. At lower salinity levels, the range of *b_pn_* tested was higher to induce no response. In addition the maximum soil salinity considered was largely lower than the range of the threshold of salinity effect on photosynthesis tested in the simulation, suggesting that the varieties used in this study were likely optimized for photosynthesis sensitivity to salinity.

## 4 Discussion

### 4.1 Evidence of model improvement with a simplistic approach

An approach involving more process-based algorithms than previous approaches to crop salinity-response simulation was used to account for rice crop responses to salinity in the upgraded version ORYZA v3 model and its integrated version within the cropping systems model APSIM-Oryza. Simultaneously, we achieved integration of a physiologically-sound mechanistic description of rice crop responses to salinity, which varies dynamically during the growth period of the crop (Lutts et al., 1995) while avoiding the need for an overly complicated description of soil salinity dynamics. Two components of salinity were comprehensively considered in our approach: (i) osmotic stress, described using a common equation to convert soil salt concentration into soil osmotic potential, and (ii) sodium toxicity, a long-term response represented by a critical level of salinity to cause 50% decrease in plant transpiration and photosynthesis. These two key processes (photosynthesis and transpiration) were used to differentiate the effect of salt accumulation in the plant on biomass production and grain yield. An approach taken for the tomato model by Karlberg et al. (2006) has also used the same approach including respiration increase with salinity and photosynthesis decrease. However, quantification of respiration over the entire crop has limitations when it comes to model validation and it did not account for variability among varieties.

Our daily time-step and in-season dynamics approach provided a more realistic description in achieving a good compromise between scientific rigor and usability. With a seasonal approach, an overestimation of the stress was obtained in a previous attempt using the ORYZA2000 rice model (Tuong et al., 2003). The model APSIM has also previously been applied in simulating salinity effect on wheat crops using the water uptake limitation approach. The parameters used were soil and crop dependent, limiting its application to other crops (Hochman et al., 2007). Biomass production is reduced then from its maximum under non-stressed conditions by multiplying a water stress factor that accounts for the osmotic stress caused by the increase of salt concentration in the soil. This stress factor depends on the dynamics of soil salinity during crop growth. Osmotic stress and salt accumulation toxicity were suggested to have a chronological occurrence inside the plants, which both models must capture over time as suggested by Munns et al. (1995). Furthermore, our representation would be valuable when considering the interaction of salinity with water deficit driven by vapour pressure deficit in salt-affected areas of drier zones (Asch et al., 1995). At higher salinity levels, osmotic stress is likely the dominant factor driving the detrimental effect on rice crop growth (Castillo et al., 2004). Thus, the improved models were demonstrated to capture this effect at acceptable performance in simulating the salinity effect on rice production across the gradients of salinity covered within the field experiments studied while maintaining their accuracy in simulating rice production under non stressed conditions (Figs. 5a & 6a).

ORYZA v3 and APSIM-Oryza use a photosynthesis-based approach to simulate daily crop growth that is limited by environmental factors such radiation, temperature, water and nitrogen stress (Bouman et al., 2001, Keating et al., 2003). We have now considered soil salinity in addition to these factors, using an approach which represents its impacts directly on photosynthesis and indirectly through water uptake and transpiration throughout the different stages in the rice crop cycle (Fig. 1). We hypothesise that these effects of salinity represent generic patterns, which make our methodology potentially applicable to models covering different crops and using different approaches, may it be functional (e.g. DSSAT, Jones et al., 2003; APSIM crops, Keating et al., 2003) or mechanistic (e.g Gecros,Yin and van Laar, 2005).

The approach detailed in this paper could then offer wider applicability in addition to its initial objective of modelling rice production in salt affected areas.

### 4.2 Model calibration and validation

The calibration procedure was particularly important in this model development. The relevant parameters in ORYZAv3 and APSIM-Oryza were not calibrated for any response to salinity. Only phenology parameters were calibrated. The salinity-response parameters used were from previous greenhouse studies for the three varieties used (Radanielson et al., 2017). The model outputs presented an RMSE_n_ between simulated and observed values ranging from 14.9 to 34.2% under non-stressed and stressed conditions for different seasons (Table 3). Phenology variables are inputs for each variety, environment, and management practice (GxExM). With crop phenology as input parameters, ORYZA v3 and APSIM-Oryza were shown to adequately simulate observed yields, with errors within the range of observed uncertainties from field experiments. Under saline conditions, crop growth slowed down, similar to drought stress, resulting in delayed phenology during the vegetative stage. In the reproductive stage, crop development was accelerated with severe salinity, while it decelerated with lower stress. This process was found to be related to a source-sink relationship during the grain-filling period.

The use of salt-related parameters for phenology allowed the representation of changes in biomass allocation between crop components in both models (stems, leaves, panicles) as partitioning also varies with phenology. Therefore, part of the salinity effect has been described through the input parameters for phenology, which have been calibrated for each level of salinity in the simulations. Both models were able to acceptably represent the crop salinity response, including the variation of crop phenology parameters. The potential for improvement in the process description exists and could be considered in addition to the present approach. The simplicity of the procedure gives the advantage of simple parameterization corresponding to the objective of the model application: a tool that allows the simulation of salinity effects on rice crop production without significantly increasing the number or complexity of input parameters and without adding overbearing complexity to data collection protocols for different levels of users.

### 4.3 Model performance

Both models were able to represent rice biomass production and yield under salt-affected conditions with RMSE values falling within the range of the standard deviation of the measured yield in the field (Table 3), thereby, indicating acceptable model performance (Gaydon et al., 2017). The range of RMSE for simulated grain yield achieved in our validation simulations was in the common range reported for other applications of the ORYZA2000 model in non-saline conditions (Bouman and van Laar, 2006; Belder et al., 2007, Boling et al., 2011, Sudhir-Yadav et al., 2011). It is important to note that the performance of the APSIM model also included daily simulation of the soil salinity dynamics, indirectly coming from applied management details (irrigation timing, amounts, and salt content), whereas ORYZA v3 used direct input of daily soil salinity values. Both models presented a close level of accuracy in simulating crop growth and grain yield. This suggests that despite the additional layer of uncertainty from simulating soil salinity, APSIM-Oryza is rendering simulations as reliable as ORYZA v3 which directly uses measured soil salinity as input. Such difference between the two models allows a distinction between their use and strength. ORYZA v3 would be most suitable for scenarios analyses under known salinity conditions and sites where adapted crop management and variety will be tested to reach desirable productivity. APSIM-ORYZA, on the other hand, would be more powerful in crop management combination assessments, management optimizations to maintain soil salinity below a desirable level, or production variability assessments with changing soil salinity levels under different potential management strategies (for example irrigation scheduling). Both models are useful for scenario analyses on water quality management and environmental conditions such as climate change.

Simulated rice yield decrease by both models is consistent with reported trends in rice responses to salinity (Van Genuchten and Gupta, 1993). Accuracy in the sensitive stage was achieved because the dynamics of salinity throughout the different stages of rice crop growth were accounted for. This advantage could be used to investigate crop management options which could be made available as targeted recommendations (such as irrigation strategies) to reduce the salinity effect on rice yield instead of taking a global approach with excessive water use for leaching requirement (Corwin et al., 2007). Other effects of salinity on rice developmental stage, biomass partitioning and yield components were not explicitly represented in the model, although these contribute significantly to the rice crop yield simulation. The level of accuracy of the models did however indicate an accounting for these effects through the modelling approach used − for instance through reduction of available biomass for grain filling resulting in reduced yield. The overestimation of yield under severe stress suggested scope for improvement by considering such stress factors at different crop stages and on harvest index.

### 4.4 Perspectives on future model use and application

The modified versions of ORYZA v3 and APSIM-Oryza demonstrated an acceptable ability to represent rice biomass and yield production under salt-affected soil conditions. This is a pioneering work in developing a reliable research tool to address salinity constraints that affect most of the irrigated rice-growing zones in coastal areas of South and Southeast Asia (Ismail et al., 2007, Radanielson et al., 2017). Both models could be used to facilitate reliable assessment of rice varieties, optimization of crop scheduling, irrigation and agronomic management, and identification of adaptive traits and adaptive crop management strategies for rice production in salt-affected areas. As an example, various potential cropping patterns and efforts to intensify production in the saline-affected coastal zones of Bangladesh could be reliably evaluated. Among possible applications of the improved rice models also include assessment of varietal tolerance to salinity as currently performed with drought-tolerant varieties (Li and Wassmann, 2010). The contribution of varietal-response-related parameters to yield variability can be estimated and their corresponding relevance evaluated as breeding traits (Radanielson et al., 2010). The genotypic parameters representing salinity response in the models represented a quantification of genotypic variability in salt tolerance expressed at the plant level. These are then a summary of different processes occurring at organ, tissue and cell level which would require further investigation to allow linking these parameters to genetic information relevant for breeding purposes.

## 5 Conclusion

Algorithms accounting for daily rice crop responses to salinity were integrated into ORYZA v3 and the APSIM-Oryza rice model within the cropping system modelling framework APSIM. New functions derived from the physiological quantification of rice responses to salinity were used. Genotypic variability in crop responses to the two major effects of salinity on crop growth and yield − osmotic stress and sodium ion toxicity − were accounted for. Both models were able to accurately simulate rice production (yield, biomass, and LAI) under different levels of soil salinity, with APSIM-Oryza reliably simulating the soil salinity dynamics directly in response to applied management. Potential opportunities remain for improved representation of salinity effects on rice phenology and biomass partitioning. However, as a result of the model improvement outlined in this paper, both models now represent reliable and improved scientific tools for researchers to address the challenges in salt-affected rice growing areas. The model improvement methodology we have followed may also suggest a path for improvement of other crop models in response to soil salinity − regardless of whether they simulate photosynthesis directly (like ORYZA v3 and APSIM-Oryza) or use a stage-related radiation use efficiency approach.
