# Supplementary material for: Modeling salinity effect on rice growth and grain yield with ORYZA v3 and APSIM-Oryza
Source: Eur J Agron. 2018 Oct;100:44–55. doi: 10.1016/j.eja.2018.01.015 (PMC7729823; doi:10.1016/j.eja.2018.01.015)
Supplement: Supplementary file 1 [file mmc1.docx]

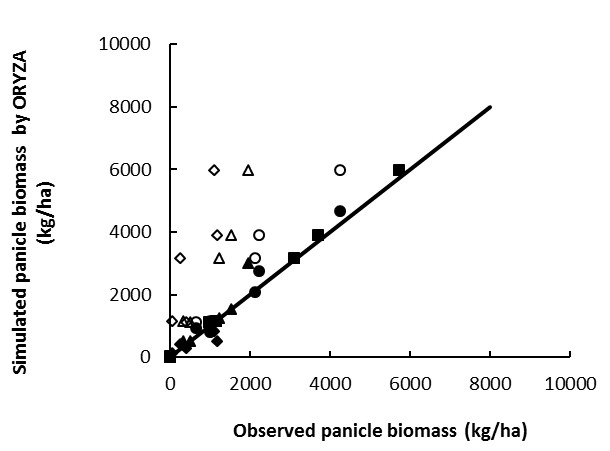

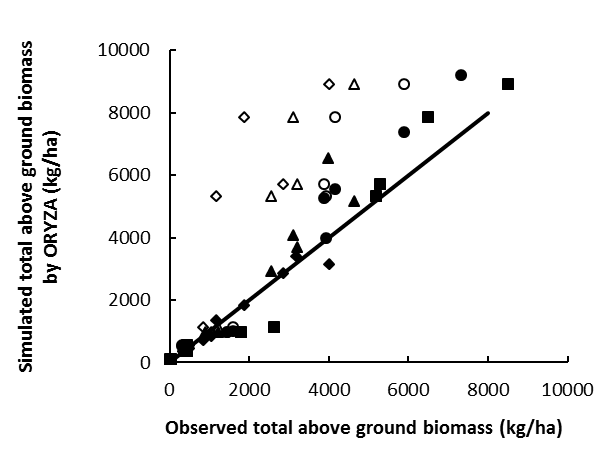

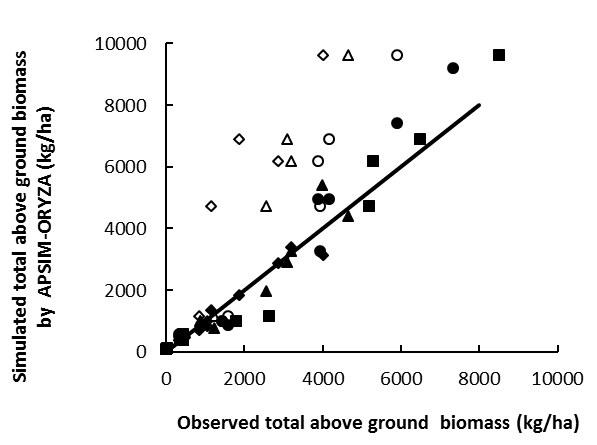

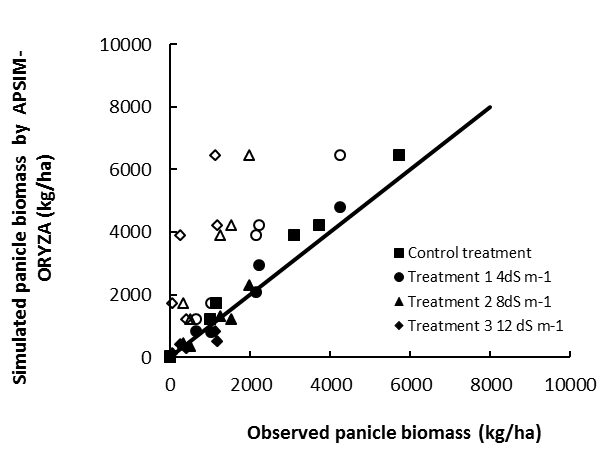


a)

b)

c)

d)

**Supplementary Figure.1** Simulated above-ground biomass and panicle biomass by the original and modified models versus observed data for the variety IR64 in salt stressed conditions. The observed data presented are average measurements from 3 replications in experiments 1 & 2. Simulated data are obtained from the ORYZA v3 model (a, b) and APSIM-Oryza (c, d), considering salinity conditions of the experiments 1 & 2. Open dot points were simulated data from the original model, complete black dot points were simulated data using the modified models accounting for salinity effect. Lines present the linear relationship 1:1 between simulated and observed values with intercept at 0.
