# Supplementary material for: Modeling salinity effect on rice growth and grain yield with ORYZA v3 and APSIM-Oryza
Source: Eur J Agron. 2018 Oct;100:44–55. doi: 10.1016/j.eja.2018.01.015 (PMC7729823; doi:10.1016/j.eja.2018.01.015)
Supplement: Supplementary file 2 [file mmc2.docx]

Supplementary Table 1. Statistical parameters of the comparison between observed and simulated data for the variety IR64 from expt 1 & 2, using original models (V.O) and modified models accounting for salinity (V.S). R^2^, coefficient of determination; RMSE, Root mean square error in kg/ha; RMSE_n_, Root mean square error normalized by the mean of the observed measurements (percentage).

| **Model** | **Version** | **Total above ground biomass**  **(WAGT, kg/ha)** | | | | | **Dry weight of storage organs**  **(WSO, kg/ha)** | | | |
| --- | --- | --- | --- | --- | --- | --- | --- | --- | --- | --- |
|  |  | Slope | | R2 | RMSE | RMSE_n_ | Slope | R2 | RMSE | RMSE_n_ |
| ORYZA V3 | V.O | 1.31 | 0.72 | | 2582 | 106.7 | 1.19 | 0.65 | 1476 | 149.2 |
|  | V.S | 0.83 | 0.95 | | 767 | 31.7 | 0.92 | 0.97 | 263 | 26.6 |
| APSIM-ORYZA | V.O | 0.53 | 0.73 | | 2704 | 111.7 | 0.49 | 0.65 | 1754 | 177.4 |
|  | V.S | 1.14 | 0.95 | | 723 | 29.8 | 0.86 | 0.98 | 314 | 31.7 |
